# Supplementary material for: EnzML: multi-label prediction of enzyme classes using InterPro signatures
Source: BMC Bioinformatics. 2012 Apr 25;13:61. doi: 10.1186/1471-2105-13-61 (PMC3483700; doi:10.1186/1471-2105-13-61)
Supplement: Addtional file 5 — The Java code to format the data files, evaluate and predict. The file enzml_java_code.tar.gz contains the Java code used to format database data to ARFF and XML formats, to execute cross and train-test (jackknife) evaluations and to record evaluation results to database. More information is included in the readme.txt file and the Javadoc files. The code can be used with a MySQL database. To use a different database software, other JDBC drivers might be required. [file 1471-2105-13-61-S5.gz › java_code/utils/doc/test/setutils/SetTest.html]

SetTest


---


|  |  |  |
| --- | --- | --- |
| **Overview** \| **Package** \| **Class** \| **Use** \| **Tree** \| **Deprecated** \| **Index** \| **Help** | |  |
| PREV CLASS   **NEXT CLASS** | **FRAMES**    **NO FRAMES**     **All Classes** |  |
| SUMMARY: NESTED \| FIELD \| CONSTR \| METHOD | DETAIL: FIELD \| CONSTR \| METHOD |  |


---


## test.setutils Class SetTest

```
java.lang.Object
  junit.framework.Assert
      junit.framework.TestCase
          test.setutils.SetTest
```

**All Implemented Interfaces:**: junit.framework.Test

---

``` public class SetTest extends junit.framework.TestCase ```

Class

**Version:**
:   16 Mar 2011

**Author:**
:   Luna De Ferrari luna.deferrari-at-ed.ac.uk

---

**Constructor Summary**

`SetTest()`


| **Method Summary** | |
| --- | --- |
| `static Set` | `getSet(java.lang.String id, java.util.TreeSet<java.lang.String> elements)` |
| `static Set` | `getSetA()` |
| `static Set` | `getSetAB()` |
| `static Set` | `getSetABC()` |
| `static Set` | `getSetAC()` |
| `static Set` | `getSetAD()` |
| `void` | `testAddSubset()` |
| `void` | `testIsSubset()` |

**Methods inherited from class junit.framework.TestCase**

`countTestCases, getName, run, run, runBare, setName, toString`

**Methods inherited from class junit.framework.Assert**

`assertEquals, assertEquals, assertEquals, assertEquals, assertEquals, assertEquals, assertEquals, assertEquals, assertEquals, assertEquals, assertEquals, assertEquals, assertEquals, assertEquals, assertEquals, assertEquals, assertEquals, assertEquals, assertEquals, assertEquals, assertFalse, assertFalse, assertNotNull, assertNotNull, assertNotSame, assertNotSame, assertNull, assertNull, assertSame, assertSame, assertTrue, assertTrue, fail, fail, failNotEquals, failNotSame, failSame, format`

**Methods inherited from class java.lang.Object**

`equals, getClass, hashCode, notify, notifyAll, wait, wait, wait`

**Constructor Detail**

### SetTest

```
public SetTest()
```


**Method Detail**

### testAddSubset

```
public void testAddSubset()
```

---


### testIsSubset

```
public void testIsSubset()
```

---


### getSet

```
public static Set getSet(java.lang.String id,
                         java.util.TreeSet<java.lang.String> elements)
```

---


### getSetA

```
public static Set getSetA()
```

---


### getSetAB

```
public static Set getSetAB()
```

---


### getSetABC

```
public static Set getSetABC()
```

---


### getSetAC

```
public static Set getSetAC()
```

---


### getSetAD

```
public static Set getSetAD()
```


---


|  |  |  |
| --- | --- | --- |
| **Overview** \| **Package** \| **Class** \| **Use** \| **Tree** \| **Deprecated** \| **Index** \| **Help** | |  |
| PREV CLASS   **NEXT CLASS** | **FRAMES**    **NO FRAMES**     **All Classes** |  |
| SUMMARY: NESTED \| FIELD \| CONSTR \| METHOD | DETAIL: FIELD \| CONSTR \| METHOD |  |


---
